# Surgically-assisted rapid maxillary expansion (SARME): indications, planning and treatment of severe maxillary deficiency in an adult patient

**DOI:** 10.1590/2177-6709.25.3.073-084.bbo

**Published:** 2020

**Authors:** Lívia Loriato, Carlos Eduardo Ferreira

**Affiliations:** 1 Pontifícia Universidade Católica (Belo Horizonte/MG, Brazil).; 2 Universidade Federal do Rio de Janeiro (Rio de Janeiro/RJ, Brazil). Universidade Gama Filho (Rio de Janeiro/RJ, Brazil).; Universidade Gama Filho, Universidade Gama Filho, Rio de Janeiro, RJ, Brazil

**Keywords:** Maxillary deficiency, Rapid maxillary expansion, Surgical expansion

## Abstract

**Introduction:**

Maxillary deficiency, also called transverse deficiency of the maxilla, may be associated with posterior crossbite, as well as with other functional changes, particularly respiratory. In adult patients, because of bone maturation and the midpalatal suture fusion, rapid maxillary expansion has to be combined with a previous surgical procedure to release the areas of resistance of the maxilla. This procedure is known as surgically-assisted rapid maxillary expansion (SARME).

**Objective:**

This study discusses the indications, characteristics and effects of SARME, and presents a clinical case of transverse and sagittal skeletal maxillary discrepancy treated using SARME and orthodontic camouflage.

## RESUMO

**Introdução:**

A atresia maxilar, ou deficiência transversa da maxila, pode estar associada à mordida cruzada posterior, além de outras alterações funcionais, especialmente respiratórias. Em pacientes adultos, devido à maturação óssea e fusão da sutura palatina mediana, a expansão rápida da maxila precisa ser associada a um procedimento cirúrgico prévio, para liberar as áreas de resistência da maxila, sendo conhecida como expansão rápida da maxila assistida cirurgicamente (ERMAC).

**Objetivo:**

O objetivo deste artigo é discutir as indicações, características e efeitos esperados da ERMAC, além de apresentar um caso clínico de deficiência maxilar esquelética transversal e sagital, cujo tratamento consistiu na ERMAC associada ao tratamento ortodôntico compensatório.

## INTRODUCTION

Posterior crossbite (PCB) is characterized by posterior maxillary teeth positioned lingually in relation to posterior mandibular teeth, and a maxillary arch more constricted than one or both sides of the mandibular arch.^1^ In skeletal posterior crossbite, the maxillary bone is transversally narrower than the mandibular bone, which characterizes maxillary atresia, also called transverse maxillary deficiency. Its correction requires orthopedic expansion,^2^ known as rapid maxillary expansion (RME).

In adult patients, it is often necessary to combine surgical and orthodontic treatments to correct posterior crossbite in a safe, efficient and predictable way.^1^ ^,^ ^3^ Factors such as ossification of the midpalatal suture (MPS) and the structure of the zygomatic buttress prevent expansion because of increased bone resistance.^4^ ^,^ ^5^ The integration between orthodontics and surgery in the treatment of maxillary atresia is called surgically-assisted rapid maxillary expansion (SARME)^6^.

SARME is a reliable procedure for the orthodontic treatment of adult patients when it is necessary to expand the maxilla.^5^ ^-^ ^8^ Several surgical techniques to release the areas of resistance of the maxilla together with conservative procedures and with stable results have been described in the literature.^2^ ^,^ ^5^ ^,^ ^6^ All the approaches include the previous placement of a fixed expander to open the MPS after surgery. Expanders may be tooth-borne, such as Hyrax, with^9^ or without^10^ ^-^ ^12^ an acrylic splint; tissue-tooth-borne, as the one described by Haas^13^; or bone-borne, using temporary skeletal anchorage devices that have been recently described, known as miniscrew-assisted rapid palatal expanders (MARPE).^14^ ^-^ ^19^ Tissue-tooth-borne expanders often lead to inflammation and ulcerations in the palatal mucosa, which makes hygiene more difficult.^15^ Comfort, easy placement and hygiene of tooth-borne expanders without an acrylic splint make them the most often used appliances, despite the fact that they apply lateral forces to the posterior teeth and alveolar bone. As they are placed away from the center of resistance of the maxilla, they produce a lateral inclination of the maxilla instead of a parallel expansion.^5^ In contrast, bone-borne expanders, directly placed on the bone using mini-implants, apply lateral forces directly to the bone, which has better biomechanical results and reduces tooth and alveolar inclination.^5^ ^,^ ^15^ However, Bortolotti et al.^8^ conducted a systematic review and found that there is no difference in how much expansion is achieved after SARME using tooth-borne or bone-borne expanders. 

SARME is indicated in the following cases: orthopedic expansion fails or cannot be used; genuine unilateral posterior crossbite; patients with complications during purely orthopedic expansion, such as intense pain, edema and palatal lesions; craniosynostosis syndrome, in which there is premature suture fusion; preparation for orthognathic surgery to achieve dental decompensation or to promote or increase stability in cases of large (more than 7 mm) dentofacial anomalies; and absolute maxillary transverse deficiency associated with deficiency of dental arch perimeter in adults.^6^

In addition to occlusal corrections, particularly that of posterior crossbite, maxillary expansion also improves nasal breathing, because it enlarges nasal volume, lowers the palate and reduces nasal resistance.^20^ ^-^ ^24^ Magnusson et al.^25^ found that patients that underwent SARME had anterior and inferior displacement, as well as widening of the nasal soft tissues, according to CT scans. 

This surgical procedure is usually conducted in a hospital, under general anesthesia, particularly when pterygomaxillary separation is necessary, because of the risk of hemorrhage due to internal maxillary artery lesion. However, it may also be performed in the office, under local anesthesia^2^ ^,^ ^4^ ^,^ ^9^ and/or sedation.^12^

Clinical cases with a thin alveolar bone between the roots of maxillary central incisors should undergo orthodontic root separation three to four months before surgery, to minimize the risk of osteotomies between roots causing asymmetric fractures in palatal suture, as well as in the alveolar bone and alveolar crest between incisors. Asymmetrical rupture of the palatal suture may result in bone defects, gingival recession with loss of interdental papillae, loss of pulp vitality, mobility or even loss of maxillary central incisor, postoperative infection, flap dehiscence and external root resorption.^6^ ^,^ ^26^ In cases of periodontal diseases, activations should be discontinued until tissues heal.^26^ Adequate clinical and radiographic evaluation should be conducted during surgical planning for SARME, to avoid these postoperative complications.^6^

Several postoperative expander activation protocols are found in the literature. Activation may start immediately after surgery,^9^ ^,^ ^12^ or after a healing period, which ranges from three to seven days.^2^ ^,^ ^7^ ^,^ ^10^ ^,^ ^11^ Activation of a ¼ turn (0.2 to 0.25 mm) twice a day until overcorrection is the most recommended protocol.^2^ ^,^ ^9^ ^-^ ^12^ Oliveira et al.^26^ recommended a two-turn activation protocol immediately after surgery until a characteristic ischemia of the palatal mucosa and a midline diastema were observed. After three days without any activation, the screw should be activated 2/4 of a turn in the morning and again in the evening, that is, one full turn a day, until the desired result is achieved. In any case, the orthodontist should monitor progression frequently and give instructions about activations to the patient.^26^ After activations, the appliance should be stabilized and kept in that position as a retainer from 90 days^9^ ^,^ ^10^ ^,^ ^12^ to six months.^7^ ^,^ ^11^ ^,^ ^27^ Gurgel et al.^28^ found that MPS ossification was not complete up to 120 days after surgery, and optical density on digitalized occlusal radiographs of adult patients that underwent SARME was not restored. At seven months after SARME, MPS density stills has only 50% to 75% of pre-treatment values on CT scans.^14^ A transpalatal arch used for retention after SARME also does not improve dentoskeletal stability^29^.

SARME effects include an increase in maxillary alveolar width and maxillary intercanine and intermolar disatances;^3^ ^,^ ^5^ ^,^ ^11^ ^,^ ^22^ correction of posterior crossbite; reduction of palate height; significant increase in palate width;^3^ and increased maxillary arch perimeter^11^ and length.^11^ ^,^ ^22^

Expansion overcorrection is recommended, because some skeletal and dentoalveolar relapse may be expected, particularly in intercanine distances.^4^ ^,^ ^5^ ^,^ ^9^ ^,^ ^12^ ^,^ ^22^ ^,^ ^30^ In contrast, Chamberland and Proffit^27^ found that skeletal changes after SARME are stable, and relapse is mostly due to the lingual movement of maxillary first molars.

Massulo et al.^10^ evaluated patients that underwent SARME immediately after stabilization and at three months of retention, and found a significant downward displacement of the posterior maxillary area and a downward and backward mandibular rotation, as well as a tendency to return to initial position, and a lingual tipping of maxillary central incisors. There was also some slight mesiobuccal rotation and significant buccal tipping of maxillary premolars and molars used as anchorage for the expander.^30^ Byloff and Mossaz^7^ confirmed SARME promotes minimal skeletal expansion by rotation, and what in fact occurs is the lateral rotation of the two maxillary halves due to the bone and dental tipping, which are responsible for part of relapse during retention and post-retention. In a recent systematic review, Bortolotti et al.^8^ found that, although SARME is efficient to obtain expansion of the transverse dimension of the maxilla, its immediate effect results primarily from dental expansion in the molar region, and not exclusively from the transverse maxillary bone increase. 

There were no negative clinical effects on the periodontium according to CBCT scans in the study by Gauthier et al.^31^ Six months after SARME, they found a reduction in buccal alveolar bone thickness and an increase in the lingual alveolar bone thickness of most teeth, a reduction of buccal alveolar crest of canine and posterior teeth, and a tendency to reduction of the mesial interproximal alveolar crest in central incisors. 

Several authors^4^ ^,^ ^9^ ^,^ ^21^ ^,^ ^22^ reported that there is no difference between patient response to RME or SARME. Indications for each procedure should be based on patient age and, above all, on their skeletal maturation.

Janson and Silva Neto^15^ recommended RME for adult patients using expanders, but no surgery, based on studies that revealed a large variation in MPS fusion according to patient age.^32^ ^,^ ^33^ The results of this procedure are not predictable or stable, and the opening of the MPS^3^ ^,^ ^4^ ^,^ ^9^ may fail, with dental or alveolar tipping in association with little or no basal skeletal movement^9^. Activation is slower, twice a week at the most, or as much as pain allows, which may extend activation time in up to two months.^34^ The increase of the clinical crown of posterior teeth,^3^ severe pain, periodontal complications, gingival recessions,^4^ ^,^ ^9^ soft tissue necrosis, tipping and extrusion of maxillary teeth, alveolar bone tipping and uncontrolled relapse^9^ may be undesired results in these cases. In adult patients, the long-term clinical significance of maxillary expansion without surgery is uncertain and questionable^35^. However, higher costs, discomfort and risks associated with SARME, in addition to patient reluctance to accept it, have led to the development of other treatment alternatives.^15^ ^,^ ^34^

The present study describes and discusses the main aspects of SARME and illustrates them with a clinical case of an adult patient with severe maxillary deficiency, bilateral posterior crossbite and Class III skeletal and dental malocclusion. This case was presented to the Brazilian Board of Orthodontics and Facial Orthopedics (BBO).

## CASE REPORT

A 53-year-old male patient sought orthodontic treatment because he often bit his mucosa when chewing certain foods. At the time, he made it clear that he would not want to undergo surgeries or extractions, and that treatment involving two-stage surgery had already been offered to him by another dentist. His general health was good, he was allergic to sulfonamide and insect bites, and reported having undergone tonsillectomy at the age of 18 years.

**Figure 1:**
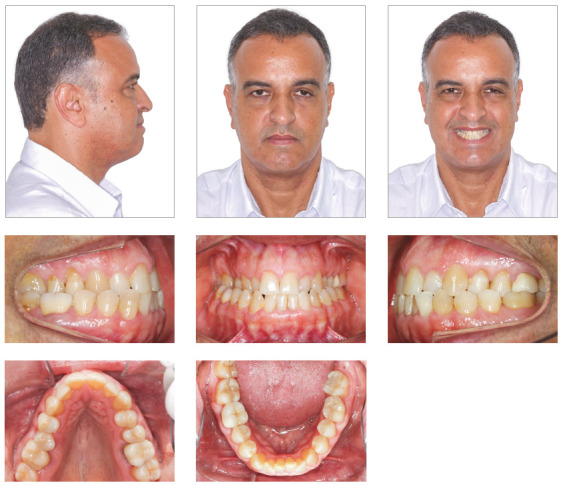
Initial facial and intraoral photographs.

Facial analysis revealed an increase in LAFH, a dolichofacial pattern, poor lip seal, concave profile, maxillary deficiency, mandibular prognathism, no gingival display on smiling and wide buccal corridors (Fig 1).

**Figure 2:**
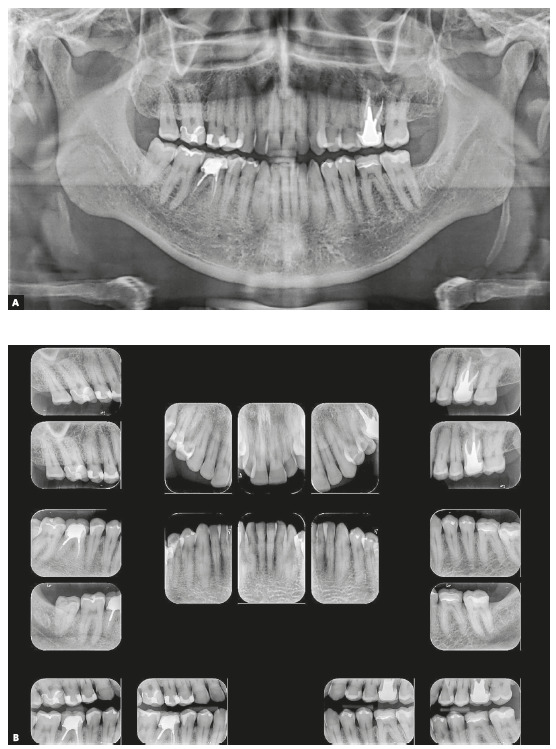
Initial panoramic (A) and periapical (B) radiographs.

Dental examination revealed a very narrow maxillary arch and an expanded mandibular arch with vertically positioned posterior teeth, bilateral posterior crossbite, non-coinciding midlines (1.5-mm maxillary deviation to the right and 2-mm mandibular deviation to the left), maxillary (2 mm) and mandibular (4 mm) crowding, asymmetric maxillary and mandibular canines and molars, Class III positioning of canines on both sides, reduced overjet and overbite, and retroclined mandibular incisors (Fig 1). Radiographs showed endodontic treatment of teeth #26 and #46, discrete generalized horizontal alveolar bone loss, generalized gingival recession, missing teeth #16, #28 and #38, and tooth migrations in the right maxillary side (Fig 2).

Functional assessment revealed a slight deviation between CR and MIP, and inadequate functional guidances. Respiratory pattern was mixed (mouth and nose breathing) and associated with nocturnal snoring. Tongue posture was low.

**Figure 3:**
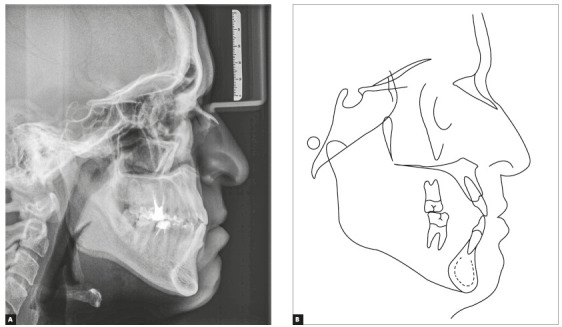
Initial cephalometric profile radiograph (A) and cephalometric tracing (B).

Skeletal analysis revealed skeletal Class III pattern (ANB = 0°) and maxillary retrusion (SNA = 80°, SNB = 80°, Wits = -2.5 mm), severe transverse maxillary deficiency, increased mandibular plane and a vertical pattern (SN.GoGn = 36°, FMA = 28°, Y-axis = 61°) (Fig 3 and Table 1).

**Table 1:** Initial (A) and final (B) cephalometric values

	Measurements		Normal	A	B	Dif. A/B
Skeletal pattern	SNA	(Steiner)	82°	80°	80°	0
	SNB	(Steiner)	80°	80°	80°	0
	ANB	(Steiner)	2°	0	0	0
	Wits	(Jacobson)	♀ 0 ± 2mm	-2.5mm	-2.5mm	0
			♂ 1 ± 2mm			
	Angle of convexity	(Downs)	0°	-1°	-2°	-1
	Y-axis	(Downs)	59°	61°	61°	0
	Facial angle	(Downs)	87°	88°	88°	0
	SN.GoGn	(Steiner)	32°	36°	36°	0
	FMA	(Tweed)	25°	28°	28°	0
Dental pattern	IMPA	(Tweed)	90°	86°	82°	-4
	1.NA (degrees)	(Steiner)	22°	27°	31°	4
	1-NA (mm)	(Steiner)	4 mm	7mm	9mm	2
	1.NB (degrees)	(Steiner)	25°	20°	15°	-5
	1-NB (mm)	(Steiner)	4 mm	6mm	6mm	0
	- Interincisal angle	(Downs)	130°	134°	135°	1
	- Apo	(Steiner)	1 mm	4mm	4.5mm	0.5
Profile	Upper lip - S-line	(Steiner)	0	-2mm	-2mm	0
	Lower lip - S-line	(Steiner)	0	1mm	1mm	0

### Treatment planning and orthodontic mechanics used

As the patient ruled out two-stage surgery and extractions from the beginning, treatment plan consisted of orthodontic camouflage combined with mini-implant anchorage in the mandibular arch for sagittal and vertical correction, and previous SARME to correct the transverse discrepancy.

Treatment objectives were: correct PCB; preserve facial characteristics, to avoid an LAFH increase and favour passive lip seal; improve smile arc, extrude and increase exposure of maxillary incisors, and increase overbite; move maxillary teeth mesially; move mandibular teeth distally and tip them lingually, to gain adequate overjet and sagittal correction. 

**Figure 4:**
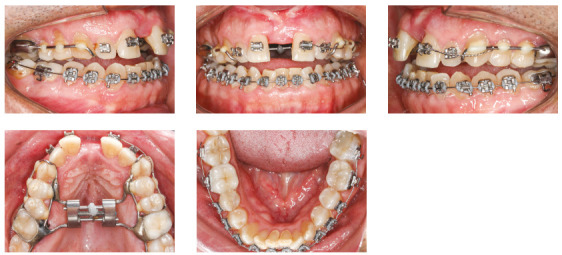
Intermediate intraoral photographs (after SARME).

Treatment started with the placement of a Hyrax expander and SARME surgery. Activation protocol was ¼ of a turn once a day for the first week, and then ¼ of a turn twice a day. However, the gingiva between teeth #11 and #21 showed signs of changes, an indication of gingival recession. At that moment, the expander was partially deactivated and the patient was asked to discontinue activations. Five days later, the patient was told to resume activations at ¼ of a turn once a day for two days, and to discontinue activation at the next day, for 10 days. This protocol was kept for 27 more days, with a favorable response of gingiva, without any recession. After PCB overcorrection, screw opening of 8.25 mm and achievement of a 7-mm interincisal diastema (Fig 4), the patient was referred to a prosthesis specialist for the restoration of central incisors, as he wished to have the diastema corrected. A 3-mm diastema was preserved to start the correction of the maxillary midline and maxillary canine asymmetry. One month after SARME, still during activation, a fixed appliance was bonded to maxillary incisors to stabilize tooth #21. It was anchored to the left side of the expander using a tie-together to ensure that only tooth #11 moved mesially. A full mandibular fixed appliance was placed (MBT prescription, 0.022 x 0.028-in slot), and leveling and alignment was performed using 0.014-in, 0.016-in and 0.018-in NiTi and 0.020-in stainless steel archwires. 

A mini-implant was placed between teeth #44 and #45, to move teeth #47 and #46 distally using a sliding-jig, and then replaced with another in the mesial aspect of tooth #46, to move the teeth that were anterior to #46 distally, until a canine Class I occlusion. 

After four months of stabilization, the expander was removed, and full fixed appliance was placed in the maxillary arch. After that, leveling and alignment was performed using 0.016-in and 0.017 x 0.025-in NiTi and 0.020-in and 0.019 x 0.026-in stainless steel archwires and elastics (more marked Class III in right side), associated with anchorage loss in the right side. In the finishing stage, a 0.019 x 0.025-in stainless steel rectangular archwire with additional bends was placed. The prosthesis specialist adjusted occlusion using selective grinding, which favored occlusal fit and vertical reduction, achieving adequate overbite. Treatment was concluded in 34 months and 28 orthodontic controls. A maxillary removable wraparound retainer was manufactured using a 0.036-in stainless steel wire. The appliance had to be used 24 hours a day for six months, for one more year during the night, and on alternate nights for six months after that. A fixed retainer manufactured with 0.028-in stainless steel wire was bonded to teeth #33 and #43. The patient was then referred to the prosthesis specialist for final restorations.

## Results

**Figure 5:**
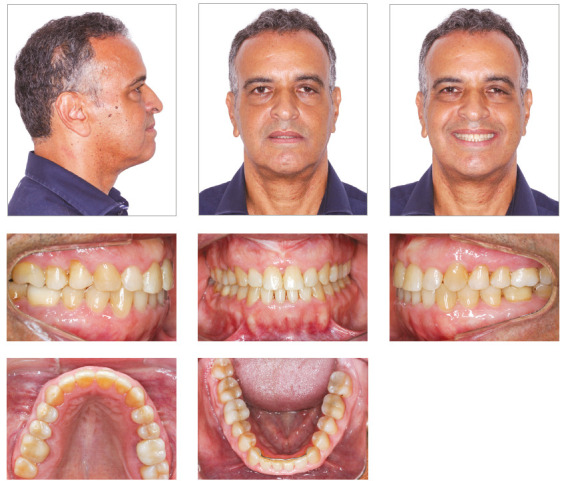
Final facial and intraoral photographs before restorative procedures.

**Figure 6:**
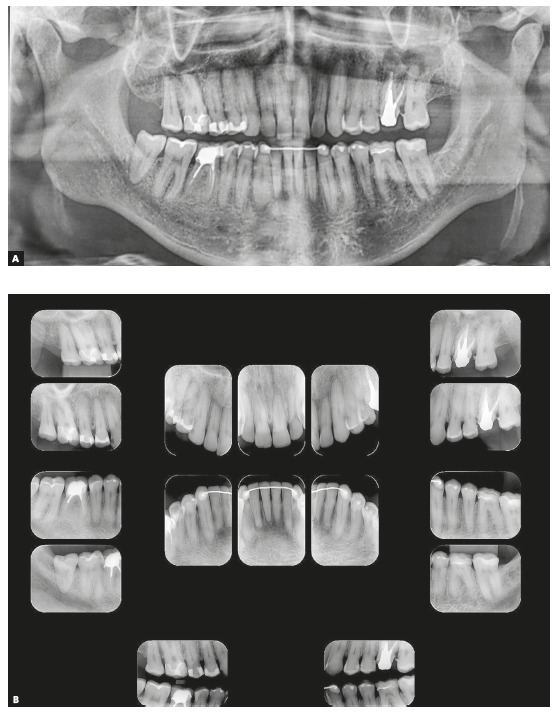
Final panoramic (A) and periapical (B) radiographs.

**Figure 7:**
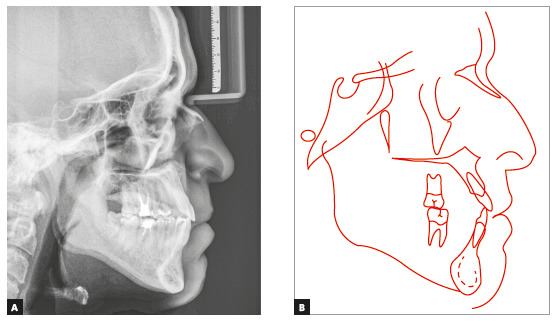
Final cephalometric profile radiograph (A) and cephalometric tracing (B).

Treatment objectives were achieved. The smile arc improved and the buccal corridors were reduced, as the distances between maxillary canines increased from 29 mm to 34 mm, and between maxillary molars, from 45 mm to 51 mm. Despite the fact that orthodontic camouflage was limited, final occlusion was highly satisfactory, with Class I molar and canine relationships, adequate overjet and overbite, and proper functional guidance free of interferences, which were a result of occlusal adjustment (Fig 5). The patient’s facial profile remained concave, but the upper lip gained better support because of the type of orthodontic camouflage conducted: maxillary incisor protrusion and mandibular incisor retrusion. Sagittal and vertical skeletal characteristics were preserved (Figs 6 and 7; Table 1). The patient reported a significant improvement in breathing immediately after SARME and chose not to undergo a speech and hearing evaluation for tongue posture, because he was satisfied with treatment results.

**Figure 8:**
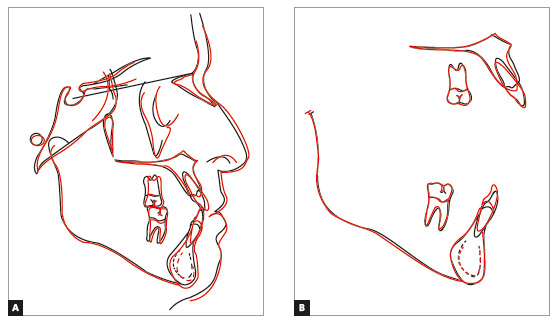
Total (A) and partial (B) superimpositions of initial (black) and final (red) cephalometric tracings.

Total superimposition of cephalometric tracings showed few changes. Partial superimposition of the maxilla showed distal movement and slight extrusion of molars, as well as extrusion and increased tipping of incisors. Partial superimposition of the mandible revealed very little movement of molars, as well as extrusion and retroclination of incisors (Fig 8).

## DISCUSSION

Maxillary expansion is a safe and efficient alternative for the treatment of maxillary deficiency^24^. In children and adolescents, the MPS is open or has very little interdigitation, and, therefore, orthopedic expansion has a good prognosis.^13^ However, in adults, resistance due to MPS fusion^32^ ^,^ ^33^ and to the structures adjacent to the maxilla, particularly the zygomatic buttress^5^ ^,^ ^19^ and pterygopalatine structures,^19^ may limit the skeletal effects of RME. For these patients, SARME is a treatment option, as it eliminates resistance in some areas of the maxilla.^2^ ^,^ ^5^ ^,^ ^6^

In the clinical case described here, severe maxillary deficiency associated with bilateral posterior and anterior crossbite, in addition to negative discrepancy in both arches, led to the choice of SARME as the first treatment option. The type of expander used for SARME may be tooth-borne, tissue-tooth-borne or bone-borne. Some authors^5^ ^,^ ^14^ ^-^ ^19^ recommend expanders in combination with temporary devices for absolute anchorage, to reduce the effects of the inclination of posterior teeth and of the two maxillary halves, as well as to achieve bone separation by lateral translation. However, according to Sevillano,^19^ randomized controlled trials have not found differences in tooth movement during expansion with or without skeletal anchorage. In the case presented here, a Hyrax expander was selected because it provides satisfactory results in SARME, good hygiene control, is easy to manufacture and has a low cost. The conventional fixed appliance was placed in the mandibular arch immediately after maxillary expansion, which ensured the buccolingual decompensation of mandibular teeth, as well as the dentoalveolar expansion of the mandibular arch. This was especially true in the region of canines, where the distance between teeth went from 23 mm to 25 mm, and of mandibular second molars, especially tooth #37, for which initial lingual tipping was increased.

Expander activation started four days after surgery, although some studies recommend immediate activation.^2^ ^,^ ^7^ ^,^ ^9^ ^-^ ^12^ Initial activation protocol had to be adapted to prevent gingival recession or dehiscence in the region, as the gingiva between maxillary central incisors underwent changes. According to some authors,^6^ ^,^ ^26^ this might have been the result of an asymmetrical fracture of the MPS in the area between incisors, but there was no radiographic control to confirm it. Surgical planning for this case did not include any recommendation of previous divergent orthodontic movement of the roots of maxillary central incisors to avoid changes in interincisal papilla, as suggested by França and Moscardini.^6^ Therefore, activations were conducted at longer intervals to ensure the preservation of the health of interincisal gingiva, as suggested by Oliveira et al.^26^

No consensus has been reached about retention time after expander stabilization, whether it should be three^9^ ^,^ ^10^ ^,^ ^12^ or six^7,11,27^ months. However, because of the patient's complaints about having to keep the appliance after four months, radiographs were obtained to evaluate new bone formation in the MPS and remove the expander. A removable appliance was then placed, both as a precaution and to ensure SARME stability. It should be used continuously for two months, and every night for one month after that, together with the conventional orthodontic appliance placed in the maxillary arch. 

Before SARME, the patient reported that his nasal breathing was not satisfactory. No specific evaluation of this function was conducted, but the patient identified a significant improvement in nasal breathing and in sleep quality immediately after SARME, which corroborates other evidence^20^ ^-^ ^24^ about the benefits of SARME for nasal breathing. 

According to McNamara,^24^ maxillary deficiency syndrome is found in about half of the patients with Class III malocclusion and skeletal maxillary retrusion, which is associated with posterior and anterior crossbite and maxillary crowding. The patient in the case presented here had maxillary deficiency, skeletal Class III malocclusion due to maxillary retrusion, according to the cephalometric analysis (Fig 3 and Table 1), posterior and anterior crossbite and severe mandibular crowding. Although ideal treatment would involve a second surgical stage to correct Class III sagittal skeletal discrepancy, as well as an extraction in the right hemimandible because of mandibular crowding and asymmetry between canines, these options were ruled out by the patient. Therefore, treatment consisted of orthodontic camouflage for sagittal correction, together with the use of intermaxillary elastics and distal movement of the right mandibular teeth using a mini-implant and a sliding jig,^36^ achieving satisfactory results. Tooth alignment, a better smile arc shape and the narrowing of the buccal corridors were fundamental to improving smile aesthetics.

RME combined with the use of mini-implant, a procedure known as MARPE, is more conservative and has lower costs and risks than SARME, and, because of that, has gained attention in the literature.^15^ ^-^ ^19^ However, it is primarily indicated for young adults in their 20s to 30s, although no age limits are found in the literature. Bortolotti et al.^8^ recommend nonsurgical RME for adult patients, because the skeletal expansion achieved with SARME is minimal and there is the risk of the morbidities inherent to surgery. However, in the case presented here, the more advanced age of the patient (53 year-old) and the severity of malocclusion favored the indication of SARME. No other treatment was considered at the time because of the chances of failure in case conventional RME or MARPE were used, as the sagittal compensation initially planned would not be achieved, and periodontal risk would be greater.

Treatment results were in agreement with changes already described in the literature: increase in maxillary alveolar width^5^ and maxillary intercanine and intermolar distances;^3^ ^,^ ^5^ ^,^ ^11^ ^,^ ^22^ posterior crossbite correction; reduction of palate height; significant increase in palate width;^3^ and increased maxillary arch perimeter^11^ and length.^11^ ^,^ ^22^ Skeletal changes achieved with SARME were stable, despite some relapse in dental expansion due to the lingual movement of the maxillary first molars, as also reported by Chamberland and Proffit.^27^

## CONCLUSION

According to the literature and the clinical case presented here, maxillary deficiency and posterior crossbite in an adult patient at an advanced stage of skeletal maturation may be efficiently corrected using SARME, with stable and satisfactory functional and aesthetic results of the skeletal, dental and smile changes.
